# Current Trends in Artificial Intelligence and Bovine Mastitis Research: A Bibliometric Review Approach

**DOI:** 10.3390/ani14142023

**Published:** 2024-07-09

**Authors:** Thatiane Mendes Mitsunaga, Breno Luis Nery Garcia, Ligia Beatriz Rizzanti Pereira, Yuri Campos Braga Costa, Roberto Fray da Silva, Alexandre Cláudio Botazzo Delbem, Marcos Veiga dos Santos

**Affiliations:** 1 Luiz de Queiroz College of Agriculture—ESALQ, University of São Paulo, Av. Pádua Dias, 11, Piracicaba 13418-900, SP, Brazil; thatiane.mitsunaga@usp.br; 2 School of Veterinary Medicine and Animal Science, University of São Paulo, Pirassununga 13635-900, SP, Brazil; brenoluis.garcia@ucalgary.ca (B.L.N.G.); ligiarizzanti@usp.br (L.B.R.P.); 3 São Paulo State College of Technology, Americana 13469-111, SP, Brazil; yuri.costa4@fatec.sp.gov.br; 4 Biosystems Engineering Department, Luiz de Queiroz College of Agriculture—ESALQ, University of São Paulo, Av. Pádua Dias, 11, Piracicaba 13418-900, SP, Brazil; roberto.fray.silva@gmail.com; 5 Center for Artificial Intelligence—C4AI, University of Sao Paulo, Av. Prof. Lúcio Martins Rodrigues, 370-Butantã, São Paulo 05508-020, SP, Brazil; acbd@icmc.usp.br; 6 Institute of Mathematics and Computer Sciences, University of São Paulo, São Carlos 13560-970, SP, Brazil

**Keywords:** mastitis, dairy cows, machine learning, detection, big data, milk production

## Abstract

**Simple Summary:**

Artificial intelligence has become essential for aiding in different knowledge domains by improving knowledge extraction from raw data and process automation. In dairy production, artificial intelligence offers promising applications in detecting and managing bovine mastitis, the most critical disease affecting the mammary gland in dairy cows, impacting milk production and profitability in dairy farms. This research evaluated the evolution of artificial intelligence applications in bovine mastitis between 2011 and 2021 using the Scopus database and the frequency of terms cited in titles, abstracts, and keywords. We selected the 62 papers that were the most relevant according to their citation index. Our results pointed out that the terms “machine learning” and “mastitis” were the most cited, with a significant increase between 2018 and 2021. There was an increase in artificial intelligence applications for bovine mastitis per country, showing applications primarily aimed at improving the current mastitis detection systems. The most cited model was artificial neural networks. We concluded that using artificial intelligence in bovine mastitis was related to mastitis detection as a vital tool to prevent this disease, considering its major impacts on dairy production and economic return.

**Abstract:**

Mastitis, an important disease in dairy cows, causes significant losses in herd profitability. Accurate diagnosis is crucial for adequate control. Studies using artificial intelligence (AI) models to classify, identify, predict, and diagnose mastitis show promise in improving mastitis control. This bibliometric review aimed to evaluate AI and bovine mastitis terms in the most relevant Scopus-indexed papers from 2011 to 2021. Sixty-two documents were analyzed, revealing key terms, prominent researchers, relevant publications, main themes, and keyword clusters. “Mastitis” and “machine learning” were the most cited terms, with an increasing trend from 2018 to 2021. Other terms, such as “sensors” and “mastitis detection”, also emerged. The United States was the most cited country and presented the largest collaboration network. Publications on mastitis and AI models notably increased from 2016 to 2021, indicating growing interest. However, few studies utilized AI for bovine mastitis detection, primarily employing artificial neural network models. This suggests a clear potential for further research in this area.

## 1. Introduction

Mastitis is the most prevalent disease in dairy cows worldwide [1] and is mainly caused by a bacterial infection [2]. This inflammation of the mammary gland manifests both in clinical (CM or clinical mastitis) and subclinical (SM or subclinical mastitis) forms [3]. In CM, there are visual alterations in the milk or mammary quarters, along with injuries in the secretory epithelium of glands. This negatively affects milk production and composition.

In some cases, mammary tissues may lose their milk synthesis capacity [4,5]. It is critical to observe that SM does not exhibit visual alterations in milk or mammary quarters. However, SM leads to a decrease in milk production, an increase in somatic cell count (SCC), a higher risk of CM, and a higher risk of early culling of the affected cows [6,7]. 

Mastitis negatively affects the profitability of dairy herds, mainly because it is one primary cause of premature culling and significantly decreases the milk yield of affected cows [1,8]. Mastitis costs are significant, representing around CAD 662 for a milking cow per year for a typical Canadian dairy farm [9]. In a study, a substantial portion of these costs (48%) was attributed to SM, while CM and the implementation of preventive measures accounted for 34% and 15%, respectively. Moreover, the average losses per case of CM were EUR 457 for Gram-negative bacteria and EUR 101 for Gram-positive bacteria [10]. Additionally, mastitis treatment is estimated to make up 80% of the antimicrobials used on dairy farms [11]. This excessive use of antimicrobials is associated with the emergence of antimicrobial resistance [12]. 

Accurate diagnosis of mastitis allows for correctly identifying the pathogen involved, which results in the prudent use of antibiotics [13] and decisions regarding herd management and cow milking routines. In this context, using sensors and new technologies that facilitate the collection of essential data and metrics for automated decision-making allows for the rapid monitoring and control of mastitis in dairy cows [14,15]. 

Due to the growing number of large dairy herds and the resulting large scale of data being produced, the use of artificial intelligence (AI) techniques and models has become essential in monitoring and predicting the occurrence of mastitis [16]. Some commonly used indicators to assess the occurrence of mastitis are the somatic cell count (SCC), electric conductivity (EC), and milk microbiology analysis [1,17]. These can be employed in AI models to guide mastitis treatment [18].

This work aimed to critically evaluate the use of AI tools for detecting, evaluating, and predicting mastitis occurrence in dairy cows. A bibliometric review methodology was used to identify the key events, journals, methodologies and models, authors, countries, keywords, and the thematic evolution of the area. 

This methodology is complemented by an in-depth analysis of the most relevant studies in this field, aiming to classify the models used, their main inputs, and the results obtained. The main contributions of this work are as follows: (i) to conduct an in-depth analysis of the state of the art in applying AI for studying mastitis; (ii) to identify the main existing scientific gaps in applying this methodology; and (iii) to help guide researchers and market agents in decision-making related to the use of AI techniques and tools for the detection, prediction, and control of mastitis in dairy cows.

The primary scientific questions addressed in this review are as follows:

RQ1: What are the primary applications of AI models to aid in the detection and the control of mastitis in dairy cows?

RQ2: What are the primary advantages and disadvantages of using AI tools for detecting, predicting, and monitoring mastitis in dairy cows?

RQ3: What are the most observed keywords, themes, and trends in using AI tools for the detection, prediction, and monitoring of mastitis in dairy cows?

RQ4: What are the primary gaps in using AI tools for detecting, predicting, and monitoring mastitis in dairy cows?

Besides the introduction, this review is organized into the following sections: Section 2 describes the materials, tools, and methods used; Section 3 describes the main results observed and discusses the most relevant topics through an analysis of keywords, thematic evolution, main countries of origin of the analyzed articles, the network of co-occurrences of keywords, and an in-depth analysis of the most relevant articles; and Section 4 concludes the present review, presenting limitations and recommendations for future works.

## 2. Materials and Methods

**Figure 1:**
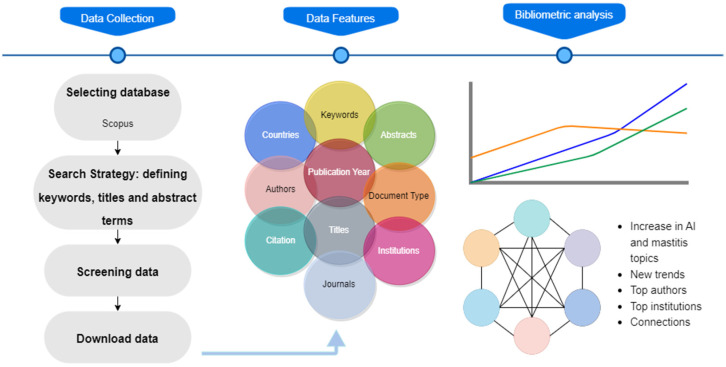
Main steps of the methodology used.

The present study used a bibliometric analysis methodology [19,20]. The following methodology steps (illustrated in Figure 1) were followed:

(a) Identification of the most frequently used keywords related to AI and mastitis. After checking the various possibilities of keywords used in previous scientific works and systematic reviews, the following keywords were used: [TITLE-ABS-KEY (mastitis) AND TITLE-ABS-KEY (dairy OR cows) AND TITLE-ABS-KEY (“artificial intelligence”) OR TITLE-ABS-KEY (“machine learning”) OR TITLE-ABS-KEY (“neural networks”) OR TITLE-ABS-KEY (“deep learning”)]. These keywords aimed to capture the main aspects related to using AI tools to control mastitis in dairy cows;
(b) Database selection for the search. The Scopus database was chosen based on its broad coverage of articles, citations, and the leading journals relevant to the analyzed theme;
(c) Definition of selection criteria for articles to be analyzed. The following criteria were used, which allowed for a better overview of the area: research area (mastitis and AI), publication period (2011–2021), and the total number of citations. The last criterion was considered the most relevant;
(d) Quantitative analysis of the evolution of keywords, leading authors, countries of origin of the publications, identification of the main types of publications (journals and scientific events), and thematic evolution of keywords. Those analyses were conducted using the ScientoPy software (version 2.1.3) [21] and the Biblioshiny R library from R (R Core Team, version 4.3.2) [22];
(e) Analysis of the co-occurrence of keywords in the paper titles and abstracts. The VosViewer software (version 1.6.20) [23] generated the network and identified the main clusters. Then, the most relevant articles for each cluster were analyzed;
(f) In-depth analysis of the ten most relevant articles identified, considering the following: the publication venue, the object of study, the main objectives, the methodology, and the main results. The AI tools identified were classified as supervised and unsupervised according to the learning method used.

## 3. Results and Discussion

Mastitis negatively affects the economic return of dairy production and is the leading cause of antimicrobial use in herds. For these reasons, mastitis control in herds is essential, and developing models to predict and monitor mastitis is increasingly necessary. Implementing AI models into databases from dairy herds could be helpful for mastitis control and high-quality milk production on dairy farms.

**Figure 2:**
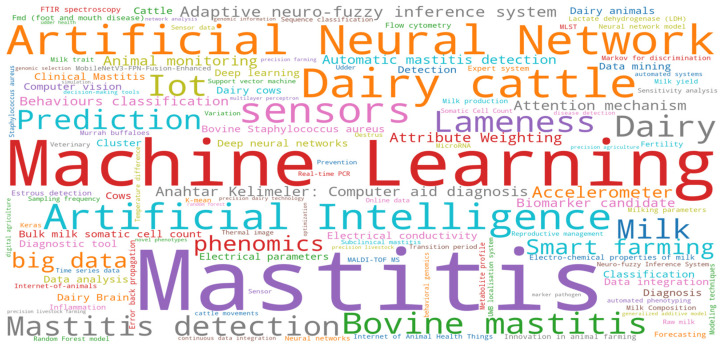
Word cloud with the main terms found in the dataset’s titles, abstracts, and keywords.

After selecting the main AI terms related to mastitis in dairy cows, 79 documents were found. Then, the filters and selection criteria previously described were applied, resulting in 55 relevant documents. The majority of these documents were scientific articles (37). The main terms found in the documents are presented in Figure 2, which comprises 33 publication venues, encompassing books, journals, and events published from 2011 to 2021.

According to the metrics generated using Biblioshiny, there was an annual growth of 15.79% in the number of documents during the period. Regarding partnerships and collaborations, 25.45% of the publications involved international partnerships.

An analysis of the word cloud was applied to the dataset. Figure 2 illustrates the most frequent keywords and terms. This approach allowed for a visual representation of the frequency of words by the font size. The word “mastitis” was the most used term, followed by “machine learning”, “artificial neural network”, “artificial intelligence”, and “dairy cattle”.

Most of the analyzed studies aimed at developing and evaluating data analysis and decision-making tools for mastitis control in dairy cows. This could explain the terms “mastitis” and “machine learning” being the most frequent words. Most of these studies used large datasets to apply analysis methods to identify mastitis’ most essential features. Different approaches were used to evaluate the use of animal data to estimate the effects of diseases in dairy production. 

In animal production, machine learning (ML) has been widely used to predict fertility outcomes, SCC, and the onset of calving, in addition to its application for epidemiological control (e.g., viruses and bacteria) [24]. ML has been used to investigate pathogens that cause mastitis and their transmission profiles. Furthermore, access to detailed milking data has provided enough data to run complex ML algorithms, enabling the diagnosis of CM and SM [16].

However, some authors point out that ML models have not yet been accurately applied to diagnosing CM at the population level [24]. The studies point out the lack of scientific evidence for the use of automatic systems for detecting CM, as none of them observed more than 80% sensitivity and 99% specificity [25,26].

**Figure 3:**
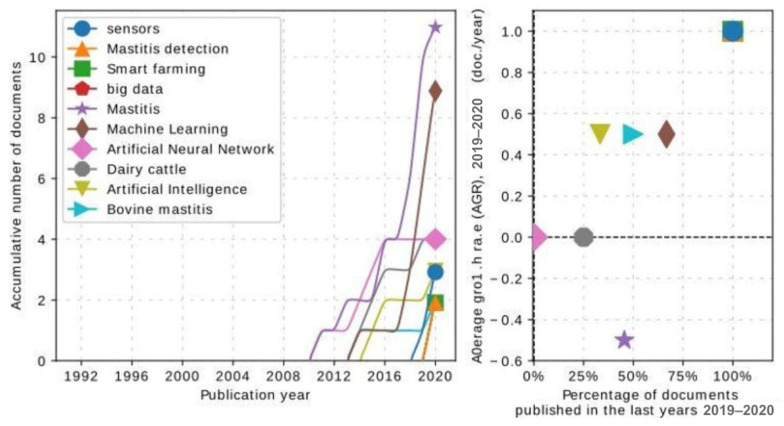
Most frequent keywords in the most relevant articles on AI and mastitis in dairy cows.

Figure 3 illustrates the trends for the ten most frequent keywords in the dataset. It shows that the most frequent terms were related to data integration and using sensors to create decision-support tools in dairy farms. Additionally, it was possible to observe a significant increase in studies evaluating AI approaches and bovine mastitis in 2016, considering the terms “mastitis” and “machine learning”. In agreement with our findings, in the review conducted by [27], the terms “dairy farm” AND “data integration” OR “dairy data management” were used to search different online scientific databases. 

Mastitis in dairy cows has been widely studied (with 7096 documents in the Scopus database at the time of writing this review) since 1917 [1]. However, considering the application of AI, the first document published in the Journal of Dairy Science was in 2000 [28]. 

In the dataset used in our analysis, considering the selection criteria applied in Scopus, we observed that the term “mastitis” appeared between 2010 and 2011. This discrepancy could be explained by the fact that only some studies may have been present in all the databases, mainly after applying the selection criteria for keywords and terms.

In the context of smart farming, using sensors allows dairy farmers to identify the most important features related to milk production volume and quality [27,29]. This increase in automated data collection has contributed to the emergence of what is known as “big data”, which has become a reality for precision agriculture. However, an extensive application in the livestock industry, including dairy farms, has not been largely explored [30].

**Figure 4:**
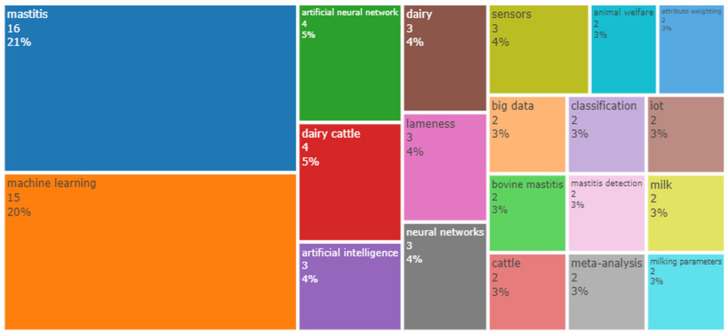
Most cited keywords in the dataset.

The keywords that were cited the most, considering mastitis in dairy cows and AI models, were mastitis and machine learning (Figure 4). This was expected after our previous analysis of the word cloud and the number of publications per year. Thus, [16,31,32,33,34] used in their keywords the term “machine learning”, pointing out that ML is the most common term to refer to the use of AI models applied for mastitis control in dairy cows.

According to the results shown in Figure 3 and Figure 4, the term “artificial neural networks” (ANN) was the most frequent ML model applied for extracting information from data. According to [14], ANNs have been widely used since 1995 in datasets from dairy herds to detect mastitis using data such as EC, SCC, lactate dehydrogenase (LDH), milk yield, udder characteristics, breed, parity, and days from calving.

According to the results shown previously, terms related to AI were frequently used as keywords, such as artificial intelligence, machine learning, artificial neural networks, and big data. This confirms the role of AI as an innovation in the milk production scenario. However, the number of scientific publications about AI and milk production was low. For instance, if we were to compare our search results to those obtained using the same terms of AI but applied to agriculture, this would result in 2480 documents.

The term “sensors” appeared consistently in all the previous figures. Figure 3 and Figure 4 show an increase in the frequency of this term, which could be explained by the increasing number of studies evaluating the application of AI models in mastitis data of dairy cows. Thus, data collection must contain reliable and integrated data, considered one of the most significant limitations in farm data analysis. While data could be manually collected in dairy farms, this approach has a high risk of obtaining heterogeneous and incomplete information [27]. An alternative to this is using smart devices connected to the internet to aid in the farm’s control systems, provided by the Internet of Things (IoT) and cloud computing [35]. 

**Figure 5:**
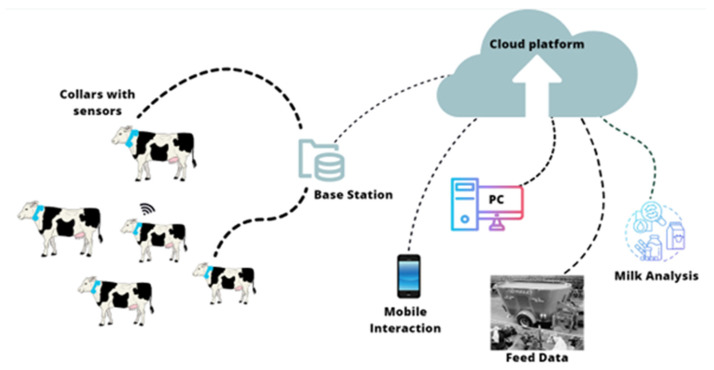
Example of IoT and cloud computing for automatic data collection in dairy farms.

Using sensors allows for the development of more structured databases. These measurement devices can be categorized into two main groups: (1) fixed-installed noninvasive devices—cameras and microphones—and (2) sensor-based animal-attached devices—ear tags, nose band sensors, and accelerometers [29]. The connection between these sensors and the system integrates highly valuable data, as described by [36] and illustrated in Figure 5.

Sensors for mastitis detection have been studied using an integrated system with measurements of milk production and other characteristics related to milk alterations, such as temperature and electrical conductivity [26]. Methods to predict the occurrence of CM using AI models allow for faster decision-making, such as the beginning of CM treatment and the segregation of cows with mastitis [37].

**Figure 6:**
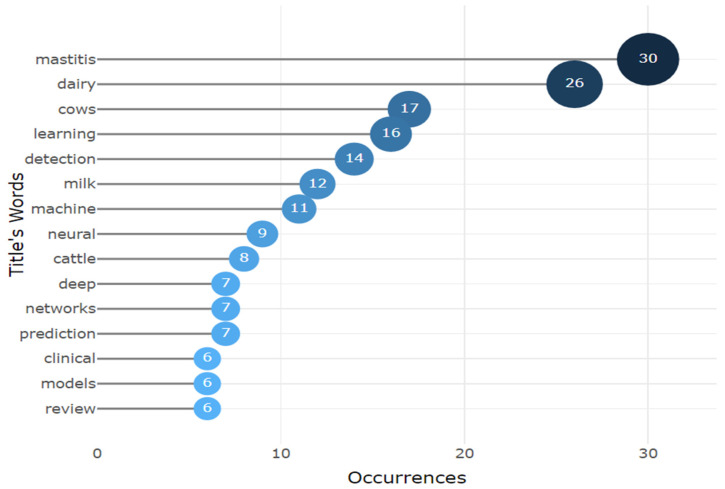
Frequency of words in the titles of the analyzed works.

Considering the occurrences of words in the titles of the works analyzed (Figure 6), “mastitis”, “dairy”, and “cows” were the most cited words, followed by terms used to describe AI models and application contexts. The word “detection” appears in position 5 of the list because of the application of AI models in the development of tools for mastitis detection.

A common practice is to process, analyze, and remove unrealistic values from the datasets obtained from farms in order to identify patterns [26]. AI models are commonly applied to transform data from dairy cows into readable values for information extraction models [33]. The variables or features can then be analyzed in terms of their importance, resulting in attribute classification, which allows for the identification of variables related to mastitis.

Moreover, using ANNs for mastitis detection was one of the primary methodologies used by the works analyzed, probably due to its better prediction and classification performances compared to traditional statistical methods [28,38]. The use of ANNs has been adopted according to the inherent ability of learning algorithms to detect patterns in data. This technique was developed as an information processing system designed to mimic how the human brain performs tasks [38] and has since been used in many different domains and contexts [39].

However, the frequency of title words related to AI terms indicates that the use of ANNs could be further explored, considering the growing demand for disease detection in dairy herds, particularly for mastitis, given its multifactorial nature.

**Figure 7:**
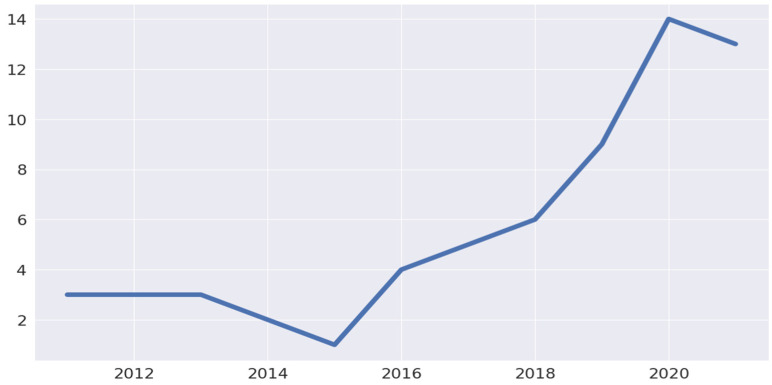
Number of publications per year in the analyzed dataset.

Based on the analysis of publications during the last ten years, AI research has significantly increased since 2016 (Figure 7). This expansion can be attributed to the increased availability of hardware, software, and data, which has enabled the production of large databases. Moreover, AI models have been applied in most fields for their learning, reasoning, and adaptation capabilities in situations where data interpretation might be a complex computational task [40].

In their review, [29] evaluated and described the use of AI in smart farming applied in animal production for various pieces of research with sensors, data processing, and transmission to aid in animal identification, behavior detection, disease monitoring, and environmental control. However, there have been no bibliometric evaluations of studies on mastitis in dairy cows and the use of AI terms. We observed that in the Scopus results, there were different terms related to dairy cattle, such as cows and milk, highlighting that a direct search of specific terms in this area could underestimate the number of publications, which could be avoided in a bibliometric evaluation.

In addition, this period coincides with the further development of deep learning algorithms, such as convolutional neural networks, which have demonstrated application in almost all areas of knowledge and research [41]. For mastitis, [42] used ANN to differentiate between healthy and sick cows, considering CM. Electrical conductivity assessments were conducted, and the authors suggested that predictive models could be improved by including additional indicators. 

Adopting an innovative approach, [14] evaluated EC, somatic cell score (SCS), LDH, and milk production as indicators of mastitis. Each indicator was decomposed into a smoothed component and a residual component to distinguish long-term trends from short-term deviations, and the residual components were used to construct a latent variable (or score) to predict the occurrence of mastitis. The authors used ANN and generalized additive models (GAMs) in the training set, resulting in similar sensitivity and specificity for both models, only decreasing the performance when omitting SCS.

**Figure 8:**
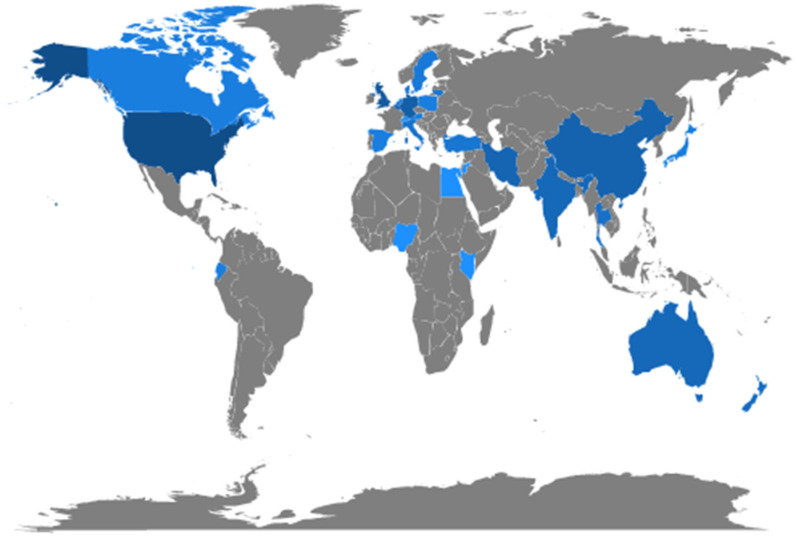
Number of publications by country. Darker colors indicate higher production.

**Figure 9:**
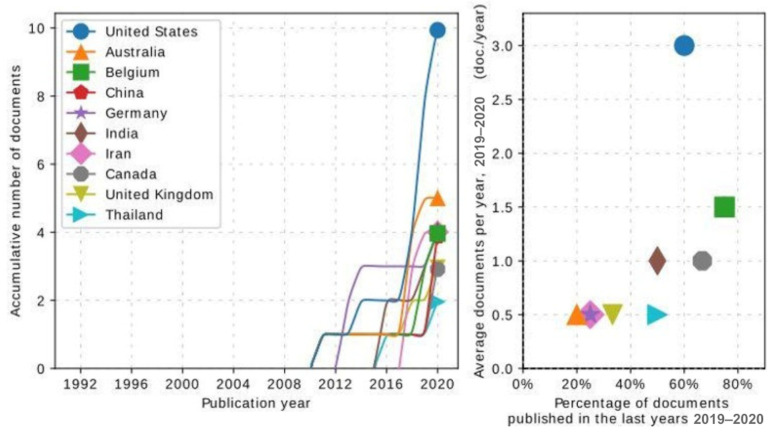
Countries with the highest number of relevant publications in the dataset.

To assess the relationship between the number of publications and the countries involved, we analyzed the countries with the most relevance in publications with terms related to our search (Figure 8 and Figure 9). The United States had the highest number of publications in recent years, which was expected as it is a developed country with a recognized advanced technology for animal production. Moreover, the US is recognized as one of the leading countries considering milk production [43].

Following the US, Australia, China, India, the United Kingdom, and Germany have considerably contributed with studies related to our search terms “mastitis” and “artificial intelligence” models. This result was expected, as these countries are important in global milk production [43]. Canada and a few other African countries had a lower frequency of studies in this area, and there was only one representative country in South America.

It was unexpected that a few countries, such as Egypt, Nigeria, Kenya, Iran, and Thailand, appear in Figure 8 and Figure 9, considering the African continent has lower milk production and low per capita consumption [43]. However, Kenya has one of the continent’s highest numbers of dairy herds [44]. As for Iran, the significant number of publications could be explained by the collaboration with Australian research teams.

**Figure 10:**
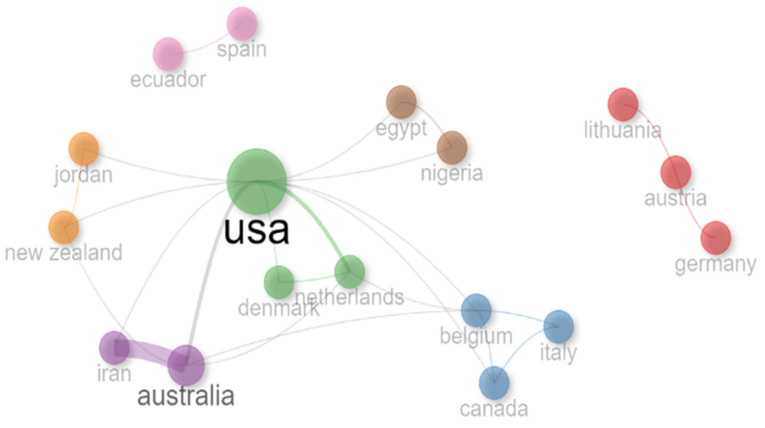
Collaboration network between countries applying AI and mastitis in dairy cows.

Figure 10 illustrates the main collaboration networks in the analyzed papers. The United States and its main collaborations are highlighted as a central node in the network due to their importance in terms of the quantity and impact of documents in the analyzed dataset. Its most significant collaborations were from Australia and the Netherlands. On the other hand, some groups had a more restricted collaboration profile, such as those from Austria, Germany, and Lithuania, which collaborated in the same way as Ecuador and Spain.

The comprehensive collaboration between the US and other countries was expected, following the previous results of most relevant studies and the number of publications per country. However, Ecuador and Spain collaborated exclusively, while a similar pattern was observed in Lithuania, Germany, and Austria. We observed that a few authors collaborated in research where they had previous contact or became new members.

The number of citations was considered to identify the ten most relevant papers. Table 1 summarizes the main information in each publication to help visualize which area AI has been used to explored mastitis in the last ten years and the most commonly used methodology.

The journals Computers and Electronics in Agriculture and Journal of Dairy Science published two relevant articles each. These results might be expected because they are leading journals publishing studies related to AI models and mastitis in dairy cows.

Considering the methodology, the most used methodology comprised ML models, with a few studies combining statistical models with ML models. The ML model with the best performance was Random Forest (RF), but we also observed that ANNs and ML are increasingly being used in data analysis.

Regarding the dataset used in the most relevant publications, milk traits such as SCC, lactose, EC, and milk yield were the most cited. SCC and SCS were the milk traits with a higher ability to aid in mastitis prediction, followed by lactose and EC. In the published studies in Table 1, we observed that the milk traits were obtained using automated recording systems, which probably allowed their use as mastitis predictors.

**Figure 11:**
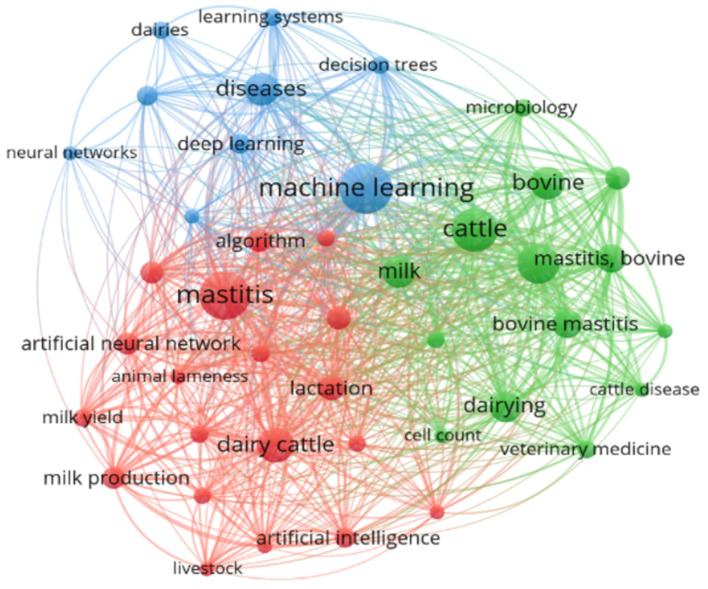
Network generated by clustering the most used terms in the titles of the articles.

Figure 11 illustrates the network generated by clustering the most frequently used terms in the titles of the analyzed works. It is possible to observe three distinct clusters, which will be further analyzed in the following paragraphs. Based on the analysis of this network, 41 keywords were identified, and the main keywords in each cluster are described in Table 2.

According to Table 2 and Figure 11, the results of grouping the keywords indicate that Cluster 1 was primarily related to AI methods and analysis approaches (machine learning, deep learning, and decision trees) and its application in milk production (dairy), as well as in disease analysis. In Cluster 3, the frequency of terms related to diseases and the health of dairy cattle (bovine mastitis and cattle disease) was observed. Cluster 2 indicates words that connect Clusters 1 and 3, and the AI methods applied for studying diseases or illnesses in dairy cows.

The first cluster, named “Machine learning and models”, demonstrates the types of models most encountered in the analysis of disease-related datasets in dairy production systems (Table 2). Using algorithms to assist in classifying animals affected by a specific disease is fundamental for predicting events related to data analysis on dairy farms [30,32,33,34].

In their study, [32] proposed a combination of two data mining tools for detecting different gene expressions and identifying the most informative genes in *Escherichia coli* mastitis. Their study analyzed data from six studies, in agreement with the following criteria: (1) “Bos taurus[organism]”, “Mastitis”, and “*Escherichia coli*” as keywords; and (2) the collection of herd information via commercial platforms, without overlapping the Affymetrix bovine GeneChipTM arrays. These data were analyzed using machine learning techniques such as attribute weighting to detect the genes with important information in the expression of *E. coli* mastitis and decision trees to combine meta-genes and bio-signature to identify biomarkers for *E. coli*.

Using the same technique of attribute weighting, [33] evaluated milk components such as milk volume, protein and lactose concentration, electrical conductivity, milking time, and peak flow as possible indicators for SM. Attribute weighting was performed in these features using the following weighting algorithms: Information Gain, Information Gain Ratio, Weighting by Rule, Weighting Standard Deviation of Attributes, Chi-Squared Statistic, Gini Index, Weighting by Uncertainty, Weighting by Relief, Support Vector Machine (SVM), and principal component analysis (PCA). The study showed that SCC was the most critical indicator for SCM, followed by lactose concentration and electrical conductivity. 

In another study, [34] combined the results from attribute weighting methods by Accuracy, Information Gain, Gini Index, and Gain Ratio with decision tree algorithms (Decision Tree, Stump Decision Tree, Parallel Decision Tree, and Random Forest). A high accuracy (90%) in predicting mastitis occurrence was obtained using the Random Forest Decision Tree with the Gini Index criterion and decision tree models identifying many patterns for SM; all cows with a low concentration of lactose and decreased milk volume had mastitis. On the other hand, cows with high lactose concentrations and low EC levels were classified as healthy cows [33].

The second cluster was named “Mastitis prediction and evaluation”. This cluster covered studies that used AI (ANN, ML) to predict diseases that affect dairy cows during lactation and that directly impact animal productivity, such as mastitis (the primary term within the cluster) (Table 2). These studies also involved decision-making compared to those determined by AI methods, considering treatment/prophylactic methods, genetic selection of important traits in dairy production, and comparisons of methods to determine the specificity and genetic differentiation of pathogens [31,45,46].

In their study, [31] compared the performance of two different ML algorithms to predict insemination outcomes in dairy cows using data such as production, reproduction, health, and genetic data stored in databases from herd management software. All data were categorized into primiparous and multiparous cows, indicating each feature’s type, means, standard deviation, and frequency of lost data.

The most used ML models to classify artificial insemination outcomes into pregnant and non-pregnant were as follows: Naïve Bayes (NB), Bayesian networks (BN), Decision Tree (DT), Bagging/Bootstrap Aggregation (BG), and Random Forest (RF). RF was the algorithm that was more effective in correctly classifying outcomes of artificial insemination. However, many misclassified results remained, probably because of the nature of data related to reproduction traits, usually those with low heritability and that are easily affected by environmental aspects. Thus, insemination outcomes can be predicted when the dataset is consistent and complete, which is a considerable limitation in most farms. 

In addition, [45] analyzed milk traits and metabolome features in Holstein cows as well as sire and farm effects in dairy farms. Milk samples were analyzed via infrared spectroscopy for the following traits: casein, protein, fat, milk yield, SCC, urea, lactose, pH, saturated and unsaturated fatty acids, and acetone. Gas chromatography–mass spectrometry (GC-MS) was applied to milk samples for analyzing their metabolome features, identifying approximately 190 metabolites.

Using two machine learning methods (RF and partial least squares—PLS), the authors predicted milk traits from metabolite patterns, with a 10-fold cross-validation for precision in prediction. They identified the metabolites with a higher correlation to traditional milk traits; uracil, lactic acid, and nine other milk metabolites are important biomarkers for SCS alterations. According to the authors, lactic acid has been cited as an important biomarker for the early identification of mastitis, and their results showed that metabolite profiles could be grouped in structures that allow for the identification of potential biosignatures.

The artificial neural network model was used in the early stages of AI applications for mastitis as a pathogen profile identification and transmission profile classification tool. The study [28] tested the use of ANN to identify four categories of mastitis-causing microorganisms: (a) contagious; (b) environmental; (c) without isolation; and (d) others. Data were obtained from field surveys and the Dairy Herd Improvement Association from cows and milk on the day of management and practices. Furthermore, milk samples were also collected for microbiological culture and identification of mastitis-causing agents.

The neural network was then developed in four stages: (1) generation of input parameters; (2) pre-processing of input data; (3) development of the neural network model; and (4) classification of the causative agents of mastitis. The data obtained were pre-processed for entry into the model, and subsequently, the microbiological identification results were regrouped into the four established categories. Two models for evaluating the NN models were developed (one with randomly selected cows and the other with cows from farms with a high prevalence of mastitis), and the models were compared to a conventional discriminatory linear model. As a result, it was observed that the classification using the neural network models had greater accuracy than that obtained by the conventional linear model.

In our dataset, [46] trained and developed the ANN model using 210 streptococci isolates obtained from bovine SM and CM and strains from blood, food, and environmental monitoring. To differentiate and identify the streptococci, samples were subject to Fourier transform infrared spectroscopy (FTIR) using the spectral windows containing information of a mixed fingerprint region of fatty acids and protein in the study as well as a region with carbohydrates, the fingerprint region with most characteristic absorbances for microorganism identification.

Moreover, matrix-assisted laser desorption/ionization time-of-flight mass spectrometry (MALDI-TOF MS) was also used to identify ribosomal proteins, RNase A, and myoglobin, important fingerprints for bacterial identification. The isolates were measured ten times to assess all biological variations, resulting in 2100 spectra for training the ANN. These data were divided into a training dataset (eight spectra of each strain), an internal validation set (one spectrum of each strain), and a test set (one spectrum of each strain). Using ANN to generate possible outcomes for mastitis Streptococci resulted in the successful identification and differentiation of Streptococci using MALDI-TOF MS and FTIR, with the second method being slightly superior. 

The third cluster is related to “Bovine and diseases”. The studies in this cluster were related to diseases affecting dairy cattle and methods for classifying and detecting parameters that indicate mastitis [14,47].

For mastitis diagnosis, variables such as SCS, LDH, electrical conductivity, and milk yield from all mammary quarters were used [14]. These variables were used to develop classifiers by selecting lactations where the cows were treated for mastitis and a control group—lactations with cows receiving no treatment. Neural network (NN) models were compared to generalized additive models (GAMs) in predicting mastitis based on SCS, LDH, milk production, and EC. The results showed that both models could accurately predict mastitis, with SCS increasing the prediction power by 5%. Therefore, both models show potential as tools for predicting mastitis in dairy cows, but further research is needed to optimize their performance and identify the most effective input variables.

To identify disease spread in dairy herds, [47] collected data on cattle movements between herds and other non-reportable local contacts between farms. The authors used data from 44 dairy herds in the UK obtained in five months to parameterize a network model of disease spread. Each farm was considered a node, and the edges represented the relationship between a given pair of farms with the potential to transmit *Staphylococcus aureus*. The study compared two time points (t1 = May 2007 and t2 = October 2007) using bulk tank milk sample results collected and analyzed for *S. aureus* through a combination of two typing techniques: a random amplified polymorphic DNA (RAPD) assay and multi-locus sequence typing (MLST).

Phenotypic identification by polymerase chain reaction (PCR) was performed in each sample positive for *S. aureus*. Farms were classified based on the presence of *S. aureus* in the bulk tank at the two time points as follows: (1) susceptible and infected, and (2) persistently susceptible, recovered, newly infected, and persistently infected. To validate the hypothesis of the transmission of *S. aureus* between farms, a random network was generated, resulting in 1000 simulations containing *S. aureus* as a fixed node. The simulations of movements between farms using actual data allowed for the description of strain spreading. It was suggested that incorporating real-world data on non-reportable contacts can improve the accuracy of network models and provide a more comprehensive understanding of disease transmission dynamics between farms.

**Figure 12:**
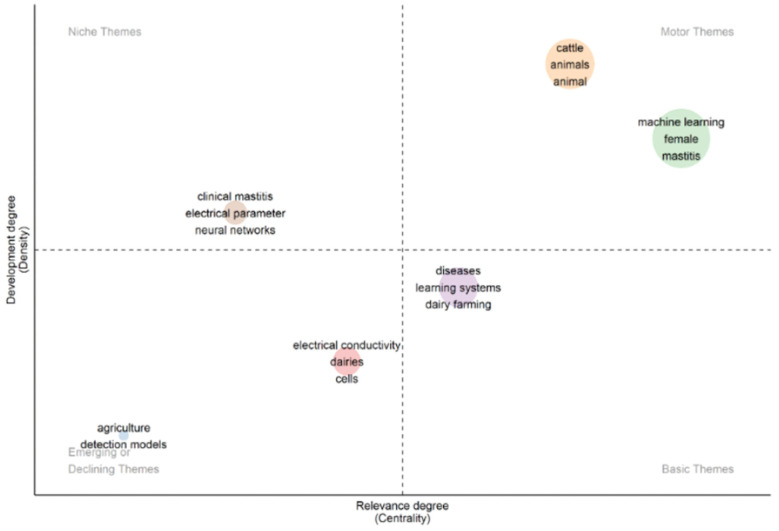
Thematic grouping of terms from the analyzed studies.

Finally, an analysis was performed considering the grouping of terms from our dataset into the following four thematic categories (Figure 12):

- Niche themes: clustering performed according to specific approaches in the articles.
- Emerging or declining themes: this cluster identified new/growing or declining themes in recent years, indicating future research needs.
- Motor themes: this cluster demonstrated the themes with greater visibility.
- Basic themes: clustering groups by terms considered basic/typical in most works.

The studies in the niche themes category were mainly related to a specific topic, with considerable frequency, although they were not very relevant for the dairy farm scenario. In the motor themes group, the studies were grouped by terms that are widely used in dairy production, such as “mastitis”, “machine learning”, “cattle”, and “animal”. As previously discussed, most of the analyzed studies used AI models for mastitis detection, which could be classified as one of the most important and cited terms. 

The basic themes group comprised terms commonly cited in studies about mastitis and AI models, which could be sorted as general or conventional terms. Considering the emerging or declining themes group, the terms “agriculture” and “detection models” were probably the least cited terms, possibly due to the nature of our search, i.e., mastitis and AI models. However, considering detection models, this could be an issue to be further investigated. 

Our results demonstrate the critical applications of AI models for mastitis, but we identified a few limitations in the reviewed studies. The limited number of farms and regions in a dataset affects the representativeness and the interpretation of results [33]. Additionally, it is possible that the results in a study may not have high accuracy once only the data from the farms included in the study are analyzed, which could be not representative. Moreover, the occurrence of intermittent excretion of pathogens by cows may not be detected in bulk milk tanks [47].

Regarding pathogen detection, the sensitivity results were successful (>93%), but there was generally a low specificity in identifying healthy cow samples [16], which is a challenge in treatment decisions. In the case of treatment decisions taken after test results, such as CMT, the decision-making depends exclusively on the input variables for this diagnosis tool, restricting the application of AI models for different scenarios [14]. In such cases, the accuracy of models could be improved by adding more variables that might explain the nature of the results.

Integrating data into large datasets is a significant challenge because the data available for evaluation is incomplete [14,31,34]. Moreover, access to these databases is a considerable limitation in cases where the data use is restricted by the owner’s permission [30]. 

Considering farm facilities, network infrastructure plays a vital role in data collection and storage. For example, network speed is affected by data size or external interferences, which can impact the performance of devices and access to databases [30]. In addition, the performance of devices such as sensors could be affected by the type of biomarker selected for detection, which could be not detected accurately [45].

In summary, the following results were obtained:

(I) The most frequent terms in the relevant publications considering mastitis and AI over the last decade were related to machine learning models, artificial neural networks, and other technologies for mastitis detection in dairy cows. The frequency of such studies has increased, especially after 2016.
(II) Developed countries were most frequently involved in studies of AI for mastitis. The United States had the highest number of relevant publications, followed by Belgium, China, and Germany. Surprisingly, Thailand and Iran were among the ten most relevant countries in AI and mastitis in dairy cows. The collaboration network between countries could have contributed to these results.
(III) After clustering the most frequent terms, we identified three clusters: the first, with terms related to machine learning and models; the second, where most of the terms approached bovine and diseases; and the last cluster accounting for the relationship among ML models to mastitis detection or prediction.

Thus, we observed that with the use of automated data and the increase in databases, there is a rising number of studies aiming to transform such data into representative information for stakeholders in milk production. However, there is a need to improve data collection by sensors and integration in automated systems to ensure a complete data analysis. Moreover, with increased data collection, it could be possible to identify the effect of different regions on milk production traits and mastitis. Therefore, improving access to these technologies, such as sensors, IoT, and cloud computing, is a promising opportunity for the dairy industry.

## 4. Conclusions

This review critically analyzed the literature on the use of AI models and mastitis in dairy cows, which is an emerging approach for analyzing large datasets in dairy farm management. These methods were successfully used for the identification of SM, mastitis pathogen identification, and for the evaluation of animal acquisition between farms, aiding in the decision-making regarding mastitis control.

A cluster analysis revealed the existence of relationships between the terms relevant to AI and mastitis, as observed in the clusters “Machine learning and models”, “Mastitis, prediction and evaluation”, and “Cattle and diseases”. After analyzing each cluster’s terms, we observed that most studies used ML and ANNs as supervised methods to diagnose and detect diseases affecting dairy cattle and milk production. 

Our study was limited to the number of relevant publications that used AI models in dairy cow mastitis studies. This highlights the nature of the subject, which is relatively recent. For future research, we recommend new studies assessing the integration of data from dairy cows and study cases to validate the results of AI models for identifying patterns of pathogens that cause mastitis and regionalization, among others. With the advancement of new applications of ML models and the integration of data for the diagnosis of mastitis, the results could be used in milk quality improvement programs as well as in animal health and drug usage in dairy farms.

## Figures and Tables

**Table 1:** Most relevant information of the ten most relevant papers in the dataset.

Results	Methodology	Objective	Citations	Journal	Reference
ML algorithms were effective in predicting pregnant or non-pregnant cows. RF was the best model in terms of classification accuracy.	ML algorithms: Naïve Bayes (NB), Bayesian Network, Decision Tree (DT), Bagging/Bootstrap Aggregation (ensemble of DT) and Random Forest (RF).	Comparison between three ML algorithms in the prediction of insemination in cows using production, health, reproduction, and genetic information.	51	Journal of Dairy Science	[31]
For accuracy, the best method was GBT (84.9%); for error classification, GBT had the lowest percentage, and the RF model had the highest percentage; for accuracy, the best model was NB (87.4%); for sensitivity, RF was the best (99.9%); for specificity, NB was the best (39.7%).	Use of data (SCC, lactose, milk volume, EC, milking time, fat, prot, and peak flow) for application of ML, DL models and statistical models (DL, NB, DT, RF, Gradient-Boosted Tree [GBT], Generalized Linear Model [GLM], Logistic Regression [LR]).	Evaluation of prediction systems to identify the best model for predicting subclinical mastitis in cattle herds based on milk composition and SCC data.	37	Computers in Biology and Medicine	[16]
The association between meta-analysis and machine learning resulted in the accurate identification of genes that can provide a biosignature for biomarkers.	Attribute weighting algorithm (AW) and Decision Tree models (DT)—Decision Tree, Random Tree, Tree Stump, and Random Forest models.	Meta-analysis of six experiments that investigated the transcriptome of mammary gland tissue after *E. coli*-induced mastitis.	37	PLoS ONE	[32]
DT Random Forest with the Gini index criterion had greater performance in mining characteristics indicative of SM (lactose, EC, and milk volume).	ML model: Decision Tree, Stump Decision Tree, Parallel Decision Tree, and Random Forest—characteristics raised for SM.	Use of DT to determine which features are detected in the detection of subclinical mastitis (SM), independent of SCC	30	Computers and Electronics in Agriculture	[33]
LDA revealed additional metabolites. RF resulted in the highest mean value of prediction for SCS (78%). PLS identified more metabolites than RF for milk traits.	Multivariate analysis methods: clustering of influencing factors, linear discriminant analysis (LDA), Random Forest, and partial least squares.	Analysis applied to determine correlations between milk metabolites and milk characteristics (casein, protein, fat, milk yield, urea, SCC, lactose, unsaturated fatty acids, pH, and acetone).	28	Journal of Dairy Science	[45]
Both Fourier transform infrared spectroscopy (FTIR) and matrix-assisted laser desorption/ionization time-of-flight mass spectrometry (MALDI-TOF MS) obtained advanced results for identifying and differentiating mastitis-associated *Streptococcus* spp.	Use of unsupervised hierarchical cluster analysis in 383 bacterial strains.	Identification system for the differential diagnosis of *Streptococcus* spp. using FTIR and MALDI-TOF/MS.	25	BMC Veterinary Research	[46]
Animal mobility between farms plays a vital role in the spread of *S. aureus*, but other contacts between (e.g., visitors) have also been correlated with strain transmission.	The network model used data from 44 farms. Nodes were defined as farms, and edges were any relationships between farms possibly affecting the transmission of the pathogen between herds.	Network-based model application for controlling the spread of *S. aureus* between farms based on animal mobility data, reports, and questionnaires from local farms	24	Epidemiology and Infection	[47]
-	Description of the main technologies involved in animal production, presenting the main discussed subjects in the big-data context extracted from animal production.	Description of opportunities and challenges in using high-throughput phenotyping analysis, big data, and other technologies related to animal production	21	Frontiers in Genetics	[30]
The application of attribute weighting models allowed for the identification of features that are related to SM—lactose was the most important variable in the absence of SCC. EC and lactose were effective for SM detection.	Data mining, statistical models, and attribute weighting algorithms evaluated the ability of several parameters to indicate the occurrence of SM, considering the presence or absence of SCC in datasets.	Use of pattern recognition model capable of identifying the best predictors for the occurrence of bovine SM based on milk composition parameters	20	Journal of Dairy Research	[34]
Both ANNs and GAMs were similar in their ability to detect mastitis, with a sensitivity of almost 75% observed for 80% of the fixed specificity. The inclusion of SCC allowed for the improvent in prediction above 5%.	Association between ANN models and generalized additive models (GAMs) were developed using a training dataset to identify mastitis based on sensitivity and specificity.	To develop and compare methods for early mastitis detection based on automated recorded data, with EC, SCS, LDH, and milk yield as possible mastitis indicators	16	Computers and Electronics in Agriculture	[14]

**Table 2:** Main terms for each cluster identified in the analyzed dataset.

Cluster and Color	Name	Keywords
Cluster 1 (blue)	Machine learning and models	machine learning, diseases, learning systems, deep learning. Others: dairies, decision trees, electric conductivity, electrical conductivity, neural networks.
Cluster 2 (red)	Mastitis prediction and evaluation	mastitis, dairy cattle, algorithm, lactation, ANN. Others: animal lameness, animal welfare, artificial intelligence, biological marker, controlled study, decision making, decision tree, livestock, milk production, milk yield, prediction, random forest, sensitivity and specificity, support vector machine.
Cluster 3 (green)	Bovine and diseases	cattle, mastitis—bovine, milk, dairying. Others: bovine mastitis, animals, bovine, cattle disease, cattle diseases, cell count, classification, microbiology, procedures, veterinary medicine

## Data Availability

The data were retrieved from the Scopus platform. However, the data of this study will be made available by the authors (corresponding author) to any qualified researcher.
